# Sorption Mechanism and Optimization Study for the Bioremediation of Pb(II) and Cd(II) Contamination by Two Novel Isolated Strains Q3 and Q5 of *Bacillus* sp.

**DOI:** 10.3390/ijerph17114059

**Published:** 2020-06-06

**Authors:** Parviz Heidari, Antonio Panico

**Affiliations:** 1 Faculty of Agriculture, Shahrood University of Technology, Shahrood 3619995161, Iran; 2 Telematic University Pegaso, Piazza Trieste e Trento 48, 80132 Naples, Italy; antonio.panico@unipegaso.it

**Keywords:** bioremediation, bioaccumulation, PTEs removal, SEM, bacterial strains, central composite design, Pb precipitation

## Abstract

The use of bacterial strains as agents in bioremediation processes could reduce the harmfulness of potential toxic elements (PTEs) from water and soil with low or even no impact on the natural ecosystems. In this study, two new metal resistant-bacterial strains (Q3 and Q5) of *Bacillus* sp. were isolated from a sulfurous spring and their potential (as pure cultures or mixed) to remove Pb(II) and Cd(II) from an aqueous matrix was evaluated and optimized using response surface methodology (RSM). The optimal conditions for Cd(II) removal from all tested strains combinations were observed at an initial pH 5, a temperature of 38 °C, and an initial Cd(II) concentration of 50 mg L^−1^, while the performance of bacterial strains on Pb(II) removal was strongly correlated to initial pH and temperature conditions. Moreover, the efficiency of bacterial strains in removing both PTEs, Pb(II) and Cd(II), from an aqueous matrix was considerably higher when they were used as a mixed culture rather than pure. According to field emission SEM (FESEM) and EDS analysis, the two bacterial strains showed different mechanisms in removing Cd(II): *Bacillus* sp. Q5 bio-accumulated Cd(II) in its periplasmic space, whereas *Bacillus* sp. Q3 bio-accumulated Cd(II) on its cell surface. On the other hand, Pb(II) is removed by chemical precipitation (lead sulfide) induced by both *Bacillus* sp. Q3 and Q5. This study discloses new aspects of Pb(II) and Cd(II) bioremediation mechanisms in *Bacillus* species that can be extremely useful for designing and operating novel PTEs bioremediation processes.

## 1. Introduction

Potential toxic elements (PTEs) such as lead (Pb(II)), cadmium (Cd(II)), copper (Cu(II)), and chromium (Cr(VI)) can generate harmful effects on ecosystems and human health [1,2,3]. Pb(II), as a hazardous metal ion it damages DNA and proteins activity; it is a teratogenic and mutagenic element and consequently causes cancer, renal failure, neurodegenerative impairment and injures the reproductive system [4,5]. Moreover, Pb(II) detrimentally affects food production and its safety because it causes oxidative stress to plants and impairs their growth [6,7]. Cd(II) is one of the most toxic elements of the Earth; it undermines plant growth, reduces food safety, and threatens human health [2]. Furthermore, Cd(II) damages kidneys and bone and indirectly causes renal dysfunction [8]. Different conventional methods are used to eliminate and reduce the toxicity of PTEs from contaminated soils and waters, including oxidation-reduction, ion exchange, neutralization, membrane separation, and electrochemical treatment methods [9,10,11,12], however, such methods are expensive and their application at large scale is not consolidated [6,13]. Besides, washing/flushing techniques have also been comprehensively investigated for the remediation of PTEs-contaminated soils [14,15]. Despite the latter techniques achieving satisfactory PTEs removal efficiency, the chemicals involved for proper treatment performance could entail a negative environmental impact due to the potential occurrence of soil properties deterioration. Bacterial strains, thanks to their resistance to metals and tolerance to extreme environmental conditions, have been widely used to perform successfully several biotechnological processes, including the removal of organic and inorganic contaminants from soil and water (bioremediation), the extraction of valuable metals (such as copper and gold) from ores (bioleaching), the improvement of mechanical characteristics of soil (biocementation), and the partial desalination of seawater [16,17,18,19]. In particular, some bacterial strains have shown promising effectiveness in bioremediation processes aimed at removing PTEs, such as Pb(II) and Cd(II), from contaminated sites [6,20,21]. Using microorganisms could reduce the cost of the remediation process and, furthermore, promote the recovery of valuable and rare metals [22,23]. Therefore, isolation and identification of new strains of metal resistant bacteria are imperative as well as valuable efforts for discovering novel and more efficient bioremediation tools aimed at removing Pb(II) and Cd(II) from contaminated sites [2].

So far, several bacterial species, such as *Microbacterium oxydans, Enterobacter aerogenes, Pseudomonas aeruginosa,* and *Bacillus cereus*, have been isolated and successfully used in PTEs bioremediation processes [2,24,25,26]. The metal resistant-bacterial strains use different mechanisms, such as extracellular as well as intracellular sequestration, and biosorption by cell surface, to reduce the toxicity of metal ions in soils and waters [6,27]. Several factors, including metal concentrations, cell physiology, and composition, as well as the structure of microbial cells, can affect the biosorption capacity of microorganisms [28,29]. Furthermore, extracellular polymeric substances (EPS) from bacteria such as glycoproteins, humic-like substances, lipids, polysaccharides, proteins, and uronic acid play a critical role in removal processes (e.g., chemical binding, precipitation process, etc.) of PTEs from contaminated environments [30,31]. In addition, different processes, including complexation, ion exchange, and surface precipitation, are known as the main mechanisms in metal ions-EPS interactions [31]. On the base of the current knowledge about the bioremediation process, extremophile environments constitute an interesting source of microorganisms well adapted to PTEs, therefore, research conducted on such environments very often ends up isolating new bacterial strains that are particularly efficient in removing PTEs from aqueous and/or solid matrices. Moreover, the removal of PTEs in bioremediation processes occurs through a wide range of mechanisms depending on the specific microorganisms involved as well as operating conditions. Furthermore, the operating conditions, as independent variables, including pH, temperature, and metal concentration, play key regulatory roles in bioremediation processes [2,13,20]. Optimizing the independent variables can improve the bioremediation efficiency and reduce costs as well as the time of the remediation treatment [32]. According to the previous considerations, this study is characterized by three main elements of novelty, as follow: (i) the isolation of new bacterial strains from an extremophile environment; (ii) the identifications of the PTEs removal mechanisms at the operating conditions when the bioremediation process achieves the highest efficiency, and; (iii) the development of an integrated experimental and numerical approach very powerful to optimize the operating conditions of the bioremediation process. Regarding this latter aspect, the response surface methodology (RSM), as a statistical method, was used to find the correlation between environmental factors and metals removal efficiency.

## 2. Materials and Methods

The whole experimental activity is composed of a chronological sequence of 4 main end to end steps, as follow:

1. Isolation and identification of the new bacterial strains;
2. Study of the critical growth conditions for the selected bacterial strains in an environment contaminated by Pb(II);
3. Optimization of the operating conditions for the bioremediation process conducted by the selected bacterial strains;
4. Study of the biotic and abiotic mechanisms involved in the bioremediation process conducted by the selected bacterial strains under the optimal operating conditions.

Each experimental step from *n* = 2 to 4 was designed according to the results obtained from the n-1 step.

### 2.1. Isolation of Bacterial Strains

Bacterial strains were isolated from the sulfurous spring of Baba Gorgor (35°17′09.8″ N, 47°54′18.9″ E) in Kurdistan, province of Iran. A volume of 10 mL of spring water was diluted in 990 mL of double sterile water, and then 300 µL of such diluted sample was cultured in Luria–Bertani (LB-Miller) agar medium (Merck KGaA, Germany) that contains tryptone (10 g L^−1^), yeast extract (5 g L^−1^), agar (12 g L^−1^), and NaCl (10 g L^−1^). Finally, the samples were incubated at 30 °C for 72 h. Eight phenotypically distinct strains on the base of morphology and color were selected and then separately re-cultured in LB agar medium containing 100 mg L^−1^ of lead nitrate (Pb (NO_3_)_2_) for screening the tolerant strains. From the eight selected strains, only two pure colonies resulted to be Pb(II) tolerant. Afterward, such strains were stored at 4 °C for molecular identification and further use.

### 2.2. Molecular Identification of Isolated Bacterial Strains

The genomic DNA of isolated bacterial strains was extracted by heat and cold cycles [2], and then samples were centrifuged (Eppendorf-5804, Germany) at 8000 rpm for 8 min, and finally, the supernatant was transferred to a new microtube for molecular identification. Using the agarose gel 1% (Sigma-Aldrich, Taufkichen, Germany) and Nano Photometer (Implen N50, München, Germany), the quality and quantity of DNA were analyzed. The partial fragment of 16S rRNA gene region was amplified by PCR with universal primers [33] ((F4: 5′-CCGCCTGGGGAGTACG-3′ and Rn2: 5′-GACGGGCGGTGTGTAC-3′). The amplified 16S rRNA region was sequenced (Macrogen Inc., Seoul, South Korea). The sequenced fragments were analyzed by a BLASTn tool (https://blast.ncbi.nlm.nih.gov/Blast.cgi) for identifying the isolated bacterial strains. The multiple sequence alignment of the isolated strains was conducted using the crustal method according to the following two parameters: the highest similarity and the highest distance. A phylogenetic tree was subsequently drawn using the neighbor-joining method provided by MEGA7 software [34]. Furthermore, the phylogenetic tree was tested using bootstrap with 1000 replicates.

### 2.3. Preliminary Study on Growth Rate of the Isolated Bacterial Strains

To determine the critical growth conditions, the pure colonies of the isolated strains were cultured in contaminated medium (i.e., Pb (NO_3_)_2_) and added to LB broth medium to reach a Pb(II) concentration of 100 mg L^−1^) at different ranges of pH (4, 5, 6, 7, and 8) as well as temperature (25, 30, 35, and 40 °C). The cultured samples were placed in an incubator shaker (KS 3000, IKA, Germany) at 160 rpm and then the bacterial growth rate was determined through optical density by spectrophotometer (UNICO 2150, China) at 600 nm for different time courses (6, 24, 48, and 72 h after incubation). 

### 2.4. Optimization of Pb(II) and Cd(II) Bioremediation Process

To optimize the Pb(II) and Cd(II) bioremediation process, the central composite design (CCD) of response surface model (RSM) was used to find out the optimal bioremediation operating conditions by varying three factors (i.e., temperature, initial pH, and initial Pb(II) and Cd(II) concentration) according to results obtained from the preliminary study on bacterial strain growth (Table 1). In brief, 400 µL of liquid media from pure cultures (Q3 and Q5) and mixed bacterial culture (i.e., obtained mixing Q3 withS Q5) were added to LB broth media according to the following operating conditions: (i) concentrations of Pb(II) and Cd(II) ranging from 50 and 200 mg L^−1^ for each metal; (ii) initial pH ranging from 5 to 8; (iii) temperature ranging from 30 to 40 °C. After 72 h of incubation, the residual Pb(II) and Cd(II) concentrations were measured by atomic absorption spectrometer (GBC, Australia), and finally, the metal removal efficiency (%) was evaluated using the following Equation (1):

metal removal efficiency = [(Ci − Cf)/ Ci] × 100(1)

where Ci is the initial concentration (mg L^−^^1^) of metal, net of precipitation occurred in growth medium solution after adding metals, and Cf is the final concentration (mg L^−^^1^) of metal.

Finally, the metal removal efficiency for Pb(II) and Cd(II) was used as a response factor in the RSM process.

### 2.5. FESEM and EDS Analyses

FESEM and EDS analysis were used to study the bioremediation mechanisms of isolated bacterial strains. For this purpose, Q3 and Q5 were separately grown at optimal conditions for Pb(II) (pH = 6.0, temperature = 35.0 °C, and Pb(II) concentration = 100 mg L^−1^) and Cd(II) (pH = 5.0, temperature = 38.0 °C, and Cd(II) concentration = 50 mg L^−1^) bioremediation process. After 48 h, all samples were centrifuged and biomass dried at 60 °C in an oven (T6120 Heraeus, Germany). Moreover, contaminated media without bacterial strain were used to perform negative control tests at optimum operating conditions. The dried samples were coated with gold. A Field Emission SEM (FESEM: Zeiss, Germany) and an Energy Dispersive Spectrometer (EDS) (Oxford Aztec1, High Wycombe, UK) analyses were used to study the topographical and elemental composition of the samples. In the EDS analysis, the elemental composition of precipitates from negative control tests was investigated using the map option while the point option was used to determine the elemental composition of the cell wall of the studied strains.

### 2.6. Data Analysis

Multiple factors (i.e., initial pH, temperature, bacterial strain) in triplicate were analyzed by ANOVA (analysis of variance) to determine the significant bacterial growth rate by using SPSS software version 17 (SPSS 2008, IBM, Armonk, NY, USA). The graphs of the growth rate profile of isolated strains were drawn based on mean values and standard division (SD) by GraphPad Prism software version 6.0 (GraphPad, San Diego, CA, USA). Design expert 10.0.7 (Stat-Ease, Minneapolis, MN, USA) was used to design the optimization process and analyze the effects of independent and dependent variables.

## 3. Results

### 3.1. Evolutionary Analysis of Isolated Bacterial

**Figure 1:**
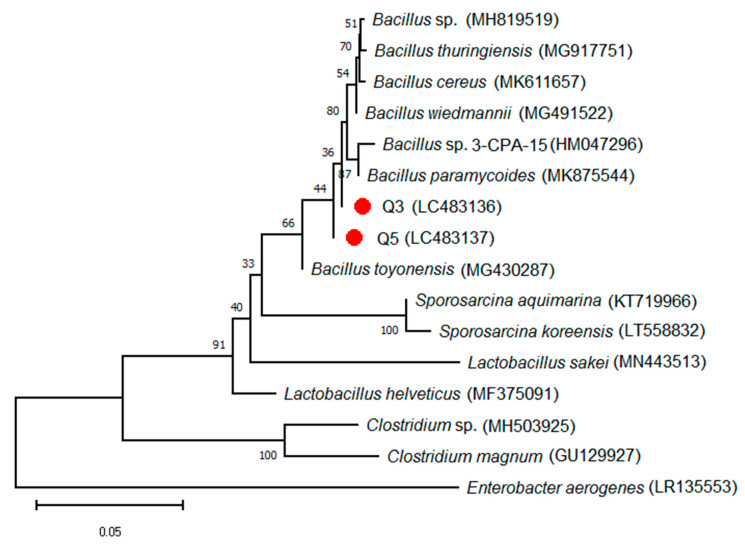
Phylogenic tree of the isolated bacterial strains Q3 and Q5 based on partial sequence of 16S region using a neighbor-joining method with 1000 bootstrap.

Based on the analysis of 16S ribosomal RNA sequencing, the isolated bacterial strains showed high similarity with *Bacillus* sp. (Figure 1). The sequences of 16S rRNA of Q3 and Q5 were deposited in the NCBI database with available accession numbers: LC483136 and LC483137, respectively. The result of the phylogenetic tree indicated that Q3 and Q5 can be considered new strains of *Bacillus* sp. as they have a genetic distance from other identified strains of *Bacillus* (Figure 1).

### 3.2. Growth Rate Profile of Q3 and Q5

**Figure 2:**
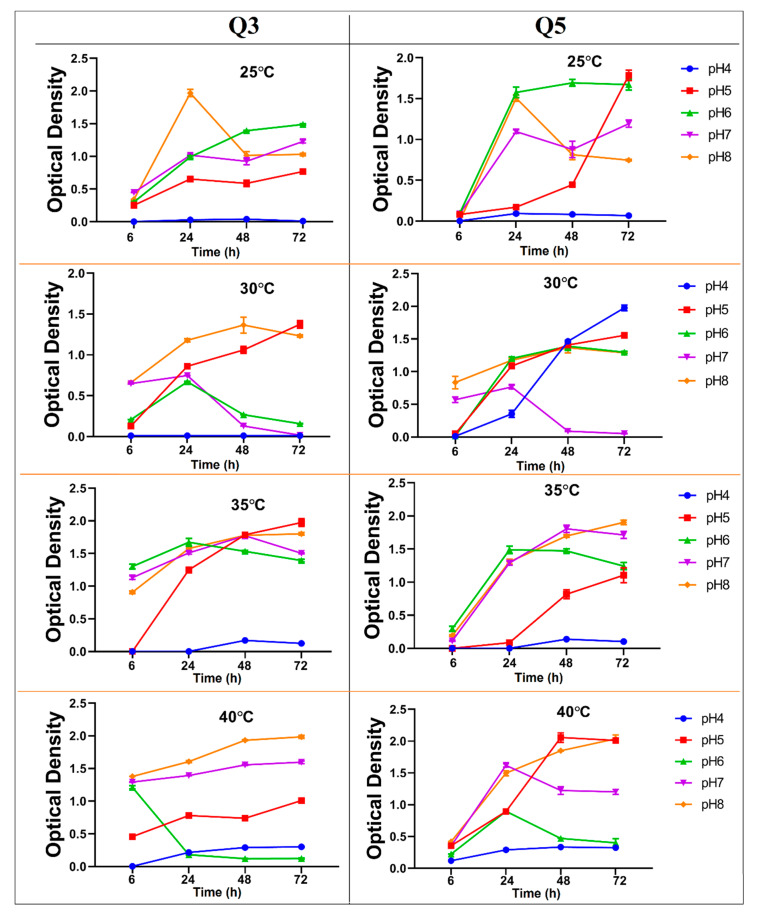
Growth rate profile of the two novel isolated bacterial strains (Q3 and Q5) at different ranges of temperature and initial pH and under contaminated condition of 100mg L^−1^ of Pb(II). Each point is the mean of three experimental replicates ± SD.

The environmental conditions such as pH, temperature, nutrient concentration, oxygen, and light intensity, as well as duration, could influence bacterial growth. The results showed that the growth of the novel isolated bacterial strains is inhibited when the initial pH is around 4 and temperature around 25 °C (Figure 2). Moreover, a significant interaction effect (*p*-value < 0.01) on the growth rate of the bacterial strains was observed between initial pH and temperature (data not shown). Bacterial strains Q3 and Q5 showed a high growth rate at initial pH 8. According to the results of the growth rate profile of the novel isolated bacterial strains, initial pH values between 5 and 8, and temperature between 30 °C and 40 °C, were selected as critical ranges for the optimization process conducted by RSM.

### 3.3. Optimization of Pb(II) and Cd(II) Removal Efficiency through RSM Using CCD

Composition of growth media, temperature, and initial pH are factors that significantly can affect the bioremediation process performance and, therefore, the optimization of these factors plays a key role in the success of the bioremediation treatment. In order to achieve the maximum removal efficiency of Pb(II) and Cd(II), temperature, initial pH, and initial metal concentration as independent variables were optimized by using the central composite design (CCD) model of RSM. Twenty experiments were conducted to evaluate the bioremediation capability of the two isolated bacterial strains tested as pure cultures as well as a mixed culture. Table 2 shows CCD design for three parameters, such as temperature, initial pH, and initial metals (i.e., Pb(II) and Cd(II)) concentration with three levels. As shown in Table 2, it was found there was very little difference between the observed and expected values, thus proving the suitability of the regression model. Moreover, the full quadratic model was used to develop a significant RSM of metals bioremediation process conducted by the two isolated bacterial strains (Equations (2)–(7)):

Pb(II) removal efficiency (%) from Q3 = −1193.25844 + 28.81298A + 214.67912B + 1.00799C − 1.01500AB + 0.00386AC − 0.055156BC − 0.29255A^2^ − 13.70162B^2^ − 0.0032638C^2^ (2)

Pb(II) removal efficiency (%) from Q5 = −672.99581 + 18.18991A + 132.55838B + 0.38660C − 0.011167AB + 0.0053967AC + 0.0115BC − 0.27393A^2^ − 10.73475B^2^ − 0.0025259C^2^ (3)

Pb(II) removal efficiency (%) from Q3 + Q5 = −1230.54922 + 45.10253A + 145.47724B + 0.78053C − 0.53867AB − 0.0026867AC + 0.01353BC − 0.58749A^2^ − 9.89212B^2^ − 0.0028511C^2^ (4)

Cd(II) removal efficiency (%) from Q3 = −342.5905 + 21.66452A + 2.74603B − 0.1429C − 0.26542AB − 0.0063754AC + 0.060909BC − 0.25364A^2^ − 0.57398B^2^ − 0.00063436C^2^ (5)

Cd(II) removal efficiency (%) from Q5 = −501.34912 + 29.86752A + 15.08243B − 0.24064C − 0.28510AB − 0.0092138AC + 0.086028BC − 0.36237A^2^ − 1.75353B^2^ − 0.00065978C^2^ (6)

Cd(II) removal efficiency (%) from Q3 + Q5 = −480.85234 + 34.12547A − 17.88676B − 0.23286C − 0.27971AB − 0.0093673AC + 0.080748BC − 0.42794A^2^ + 0.95117B^2^ − 0.00017239C^2^ (7)

In Equations (2)–(7), Pb(II) and Cd(II) removal efficiency (%) is expressed as predicted values from the regression model, whereas A, B, and C are the values of temperature (°C), initial pH, and initial metal concentration (mg L^−1^), respectively. Furthermore, the results of the analysis of variance (ANOVA) for the regression model and variables are reported in Table 3. The significance level of the coefficients was investigated according to *p*-values. Concerning the Pb(II) removal efficiency from strain Q3, A, B, C, AB, BC, A^2^, B^2^, and C^2^ were significant model parameters whereas AC was not significant (Table 3). Moreover, the regression model parameters including B, A^2^, B^2^, and C^2^ for Pb(II) removal efficiency from strain Q5 and C, AB, A^2^, B^2^, and C^2^ for Pb(II) removal efficiency from the mixed culture resulted in having a significant (*p*-value < 0.001) role on the bioremediation process. Concerning the Cd(II) removal efficiency from strain Q5 as well as the mixed culture, all terms including A, B, C, AB, AC, BC, A^2^, B^2^, and C^2^ exhibited significant effects, whereas, AB, B^2^, and C^2^ were not significant for Cd(II) removal efficiency from Q3. The R^2^ values of Pb(II) and Cd(II) removal efficiency from pure Q3 andQ5, as well as the mixed culture (i.e., Q3 + Q5), are shown in Table 3. A high value of R^2^ indicates a strong correlation between the dependent variable (i.e., metal removal efficiency) and the independent variables in the regression model. A high R^2^ value was observed for all tested cases and, therefore, the proposed model is satisfactorily validated.

### 3.4. Effect of Temperature and Initial pH on Pb(II) and Cd(II) Bioremediation Process

**Figure 3:**
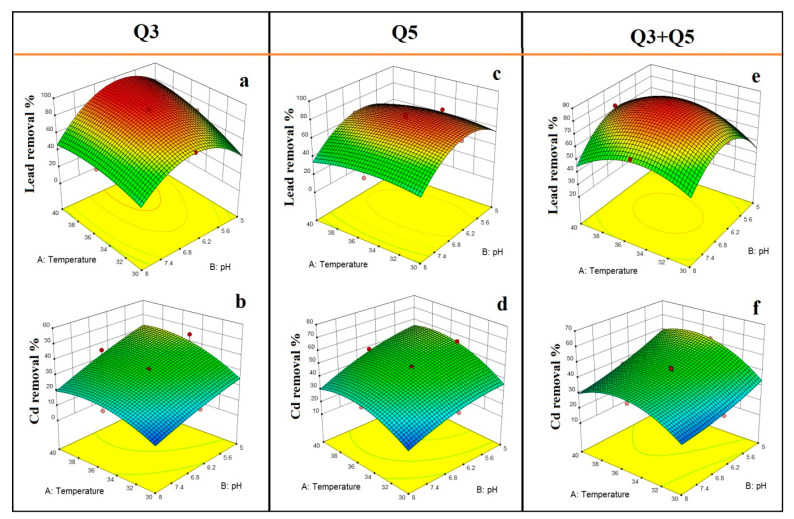
Effect of temperature and initial pH on Pb(II) and Cd(II) removal efficiency from bacterial strains Q3 and Q5 and their mixture (Q3 + Q5); (**a**) effect of temperature and initial pH on Pb(II) removal efficiency from Q3; (**b**) effect of temperature and initial pH on Cd(II) removal efficiency from Q3; (**c**) effect of temperature and initial pH on Pb(II) removal efficiency from Q5; (**d**) effect of temperature and initial pH on Cd(II) removal efficiency from Q5; (**e**) effect of temperature and initial pH on Pb(II) removal efficiency from the mixture of Q3 with Q5; (**f**) effect of temperature and initial pH on Cd(II) removal efficiency from the mixture of Q3 with Q5.

The effects of temperature and initial pH on Pb(II) and Cd(II) removal efficiency from pure cultures Q3 and Q5, as well as their mixture, are shown in Figure 3. According to 3D-surface plots, the two bacterial strains showed differences in the capability to remove Pb(II) and Cd(II) under different ranges of temperature and initial pH. The 3D plots of bacterial strain Q3 showed that initial pH and temperature play critical roles in Pb(II) bioremediation efficiency (Figure 3a). Actually, the efficiency of Pb(II) removal increased with increasing temperature and keeping the initial pH between 5.5 and 6.5. On the other hand, bacterial strain Q3 exhibited a different removal trend in a growth medium contaminated with Cd(II) where the remarkable effect of interaction, previously noticed between temperature and initial pH, was not observed. However, each factor separately affected the performance of the Cd(II) bioremediation process conducted with bacterial strain Q3, in particular, the Cd(II) removal efficiency was sharply increased under acidic initial pH (Figure 3b). Concerning the effects of temperature and pH on Pb(II) removal efficiency from strain Q5, it was noticed that initial pH was more relevant than temperature (Figure 3c). The Pb(II) removal efficiency from Q5 increased when the initial pH was set between 5.5 and 6.5. Besides, the interaction between temperature and initial pH affected significantly the Cd(II) removal efficiency from strain Q5 (Table 3; Figure 3d). Actually, at low initial pH (i.e., lower than 5.5) and high temperature (i.e., higher than 35 °C), the Cd(II) removal efficiency from Q5 increased. The effects of temperature and initial pH on Pb(II) and Cd(II) removal efficiency from the mixed culture were shown in Figure 3e,f. It was observed that the results obtained with the mixed culture were different if compared with those with pure cultures. As can be seen in Figure 3e, the Pb(II) removal efficiency from the mixed culture was increased when the initial pH was set between 5.5 and 7, and the temperature between 32 and 38 °C. Moreover, the mixed culture can remove a larger amount of Cd(II) at acidic initial pH (i.e., lower than 5.5) than pure cultures (Figure 3f). To better understand the results previously presented, it is useful to consider that the initial pH of the liquid matrix plays a key role for the metal ions availability that is higher when the initial pH is low (i.e., 5–6) and *vice-versa*.

### 3.5. Effect of Temperature and Initial Metal Concentration on Pb(II) and Cd(II) Bioremediation Process

**Figure 4:**
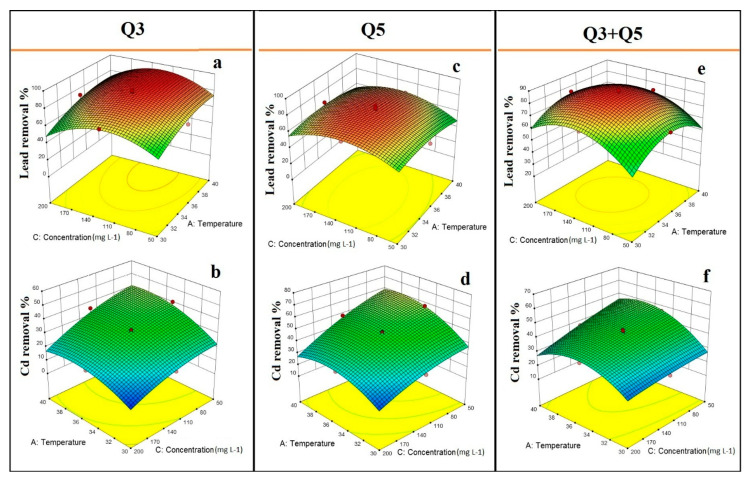
Effect of temperature and metal concentration on Pb(II) and Cd(II) removal efficiency from bacterial strains Q3 and Q5 and their mixture (Q3 + Q5): (**a**) effect of temperature and metal concentration on Pb(II) removal efficiency from Q3; (**b**) effect of temperature and metal concentration on Cd(II) removal efficiency from Q3; (**c**) effect of temperature and metal concentration on Pb(II) removal efficiency from Q5; (**d**) effect of temperature and metal concentration on Cd(II) removal efficiency from Q5; (**e**) effect of temperature and metal concentration on Pb(II) removal efficiency from the mixture of Q3 with Q5; (**f**) effect of temperature and metal concentration on Cd(II) removal efficiency from the mixture of Q3 with Q5.

**Figure 5:**
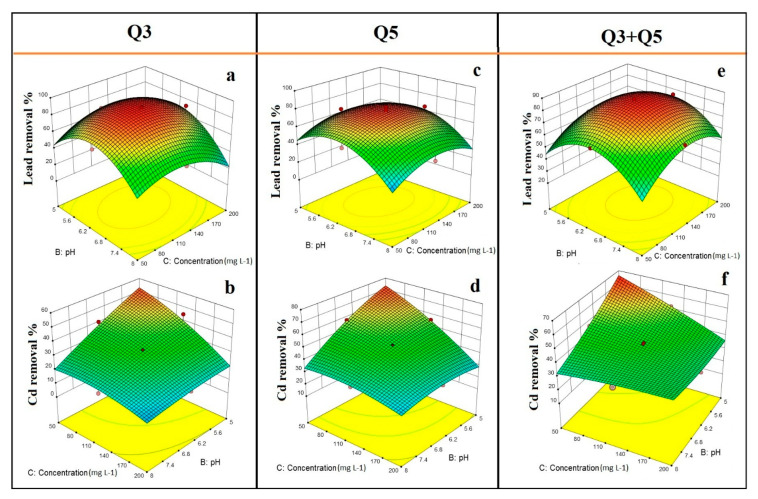
Effect of initial pH and metal concentration on Pb(II) and Cd(II) removal efficiency from bacterial strains Q3 and Q5 and their mixture (Q3 + Q5): (**a**) effect of metal concentration and initial pH on Pb(II) removal efficiency from Q3; (**b**) effect of metal concentration and initial pH on Cd(II) removal efficiency from Q3; (**c**) effect of metal concentration and initial pH on Pb(II) removal efficiency from Q5; (**d**) effect of metal concentration and initial pH on Cd(II) removal efficiency from Q5; (**e**) effect of metal concentration and initial pH on Pb(II) removal efficiency from the mixture of Q3 with Q5; (**f**) effect of metal concentration and initial pH on Cd(II) removal efficiency from the mixture of Q3 with Q5.

The interaction between temperature and initial metal concentration was not significant for Pb(II) removal efficiency from the two tested bacterial strains and their mixture. However, in increasing the temperature, the Pb(II) removal efficiency from Q3 increased (Figure 4a). A significant effect of interaction between temperature and initial Cd(II) concentration was observed for both pure bacterial strains, Q3 and Q5 (Figure 4b,d). When the initial Cd(II) concentration was low, the Cd(II) removal efficiency from pure bacterial strains Q3 and Q5 increased by increasing the temperature. The mixed culture under a specific range of temperature (i.e., 32–38 °C) and initial Pb(II) concentration (i.e., 100–180 mg L^−1^) could be more efficient in removing Pb(II) than under other operating conditions (Figure 4e). As shown in the 3D-surface plot of Figure 5f, at a high concentration of initial Cd(II) concentration, the Cd(II) removal efficiency decreased when temperature decreased as well. 

### 3.6. Effect of Initial pH and Initial Metal Concentration on Pb(II) and Cd(II) Bioremediation Process

The 3D-surface plot for interaction between initial pH and initial Pb(II) concentration showed that the Pb(II) removal efficiency from the two pure strains could be significantly higher at a specific range of initial pH (i.e., between 5.5 and 7) and initial Pb(II) concentration (i.e., 80–180 mg L^−1^) (Figure 5a,c,e). A significant effect of interaction between initial pH and initial Cd(II) concentration was observed for Cd(II) removal efficiency from pure bacterial cultures as well as the mixed culture (Table 3; Figure 5b,d,f). It was observed that the Cd(II) removal efficiency increased with decreasing both the initial Cd(II) concentration and initial pH. It can be assumed that at Cd(II) concentration higher than 50 mg L^−1^, the pure cultures were inhibited and their growth rate and bioaccumulation potential reduced.

### 3.7. Optimization of Independent Variables for Pb(II) and Cd(II) Bioremediation Process

The main purpose of the bioremediation process optimization was to find the optimal range for each independent variable involved in the Pb(II) and Cd(II) bioremediation process using the new bacterial strains of *Bacillus* sp. The results of this process are reported in Table 4. As it can be seen in Table 4, the optimal operating conditions for Pb(II) bioremediation process conducted with the pure bacterial strain Q3 were estimated as follows: (i) initial pH = 5.8; (ii) temperature = 38.8 °C; (iii) initial Pb(II) concentration = 115.4 mg L^−1^. Under these conditions, the Pb(II) removal efficiency was 93.76%. Whereas, the strain Q5 exhibited the maximum Pb(II) removal efficiency (i.e., 76.42%) at (i) initial pH = 6.2, (ii) temperature = 34.3 °C, and (iii) initial Pb(II) concentration =127.4 mg L^−1^. Finally, the mixed culture showed a different metal removal efficiency compared to pure culture, achieving a removal efficiency of 86.08% for Pb(II) at (i) initial pH = 6.5, temperature = 35.1 °C, and (iii) initial Pb(II) concentration =135.8 mg L^−1^. As it can be noted from Table 4, the new bacterial strains Q3 and Q5 are more efficient in removing Pb(II) than Cd(II). Concerning the optimal operating conditions for Cd(II) removal, all tested strains, pure cultures as well as the mixed ones, show the best performance in removing Cd(II) at (i) initial pH around 5, (ii) temperature around 38 °C, and initial Cd(II) concentration around 50 mg L^−1^. Finally, although the strain Q5, as pure culture, showed a slightly higher capability in removing Cd(II) than Pb(II), this difference (i.e., 78.0% vs. 76.4%) is too small to be considered significant. 

### 3.8. Analysis by FESEM and EDS

**Figure 6:**
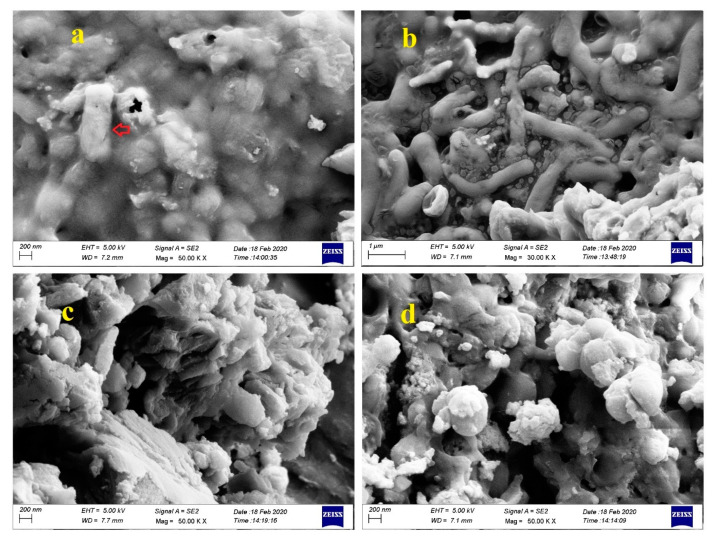
Scanning Electron Microscopy (SEM) of cell biomass of *Bacillus* sp. Q3 (**a**) and *Bacillus* sp. Q5 (**b**) at 50 mg L^−1^ of Cd(II); *Bacillus* sp. Q3 (**c**) and *Bacillus* sp. Q5 (**d**) at 100 mg L^−1^ of Pb (d). The arrow in Figure 6a represents the bio-accumulated Cd (II) in the cell surface of *Bacillus* sp. Q3.

**Figure 7:**
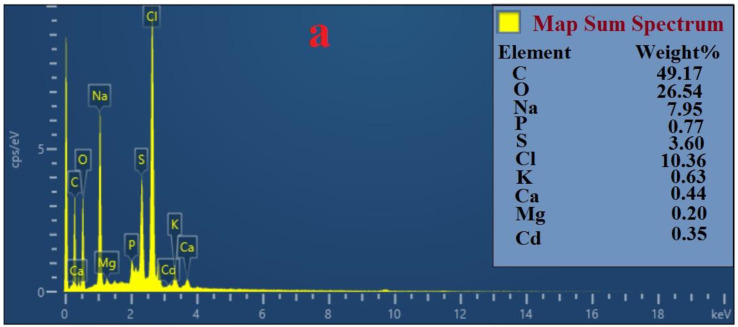

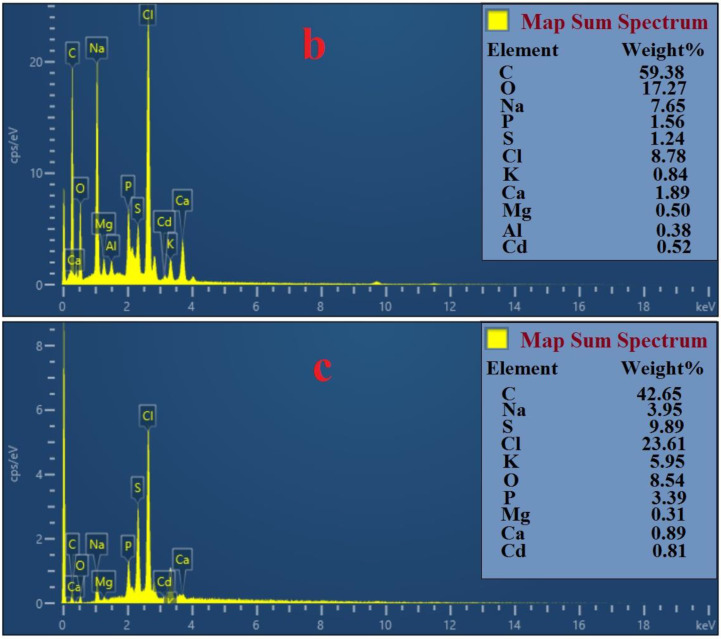
Energy Dispersive Spectrometer (EDS) analysis for elemental composition of precipitates from negative control tests (**a**), cell biomass of *Bacillus* sp. Q3 (**b**) and *Bacillus* sp. Q5 (**c**) at 50 mg L^−1^ of Cd(II).

**Figure 8:**
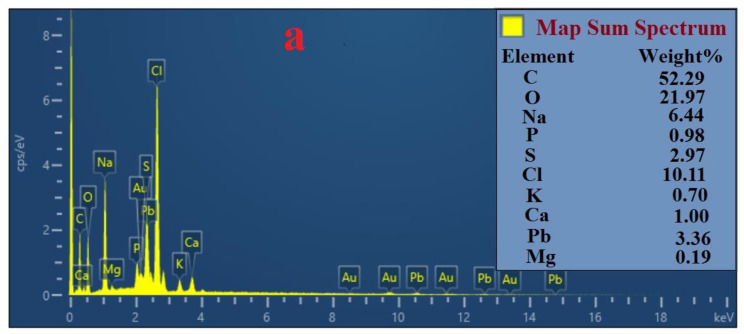

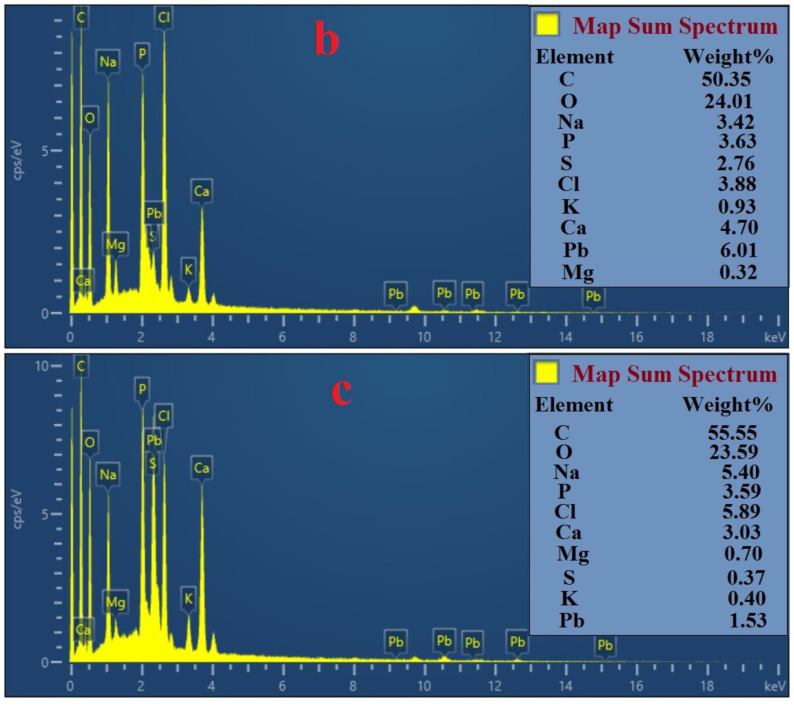
Energy Dispersive Spectrometer (EDS) analysis for elemental composition of precipitates from negative control tests (**a**), cell biomass of *Bacillus* sp. Q3 (**b**) and *Bacillus* sp. Q5 (**c**) at 100 mg L^−1^ of Pb.

To study the mechanisms of bioremediation in removing Pb(II) and Cd(II), the topographical and elemental composition of biomass forming the bacterial strains used for the experiments were analyzed through FESEM and EDS. FESEM analysis sharply indicated that the bacterial strains could absorb Pb(II) and Cd(II) into the cell wall (Figure 6). Furthermore, the EDS spectra analysis, as a useful tool to provide information about the elemental composition of bacteria biomass, revealed the presence of carbon (C), oxygen (O), sulfur (S), magnesium (Mg), potassium (K), calcium (Ca), phosphor (P), as well as Cd(II) and Pb(II) (Figure 7 and Figure 8). This result proved that Cd(II) and Pb(II) were absorbed by bacterial strains. In this study, cells of *Bacillus* sp. Q3 were covered by Cd(II) on their external surface (Figure 6a) and *Bacillus* sp. Q5 immobilized Cd(II) (Figure 6b). According to EDS spectra analysis, *Bacillus* sp. Q5 can uptake a higher amount of Cd(II) than *Bacillus* sp. Q3 (Figure 7). Besides, the percentage of P, S, K, Cl, and Cd increased in *Bacillus* sp. Q5 when compared with the results from negative control tests (Figure 7a). This aspect can demonstrate the occurrence of a precipitation process of Cd(II), induced by bacterial strains, into the bacteria cell walls as cadmium sulfate as well as cadmium phosphate. Based on FESEM analysis, Pb(II) precipitated all around the cells of both *Bacillus* sp. Q3 and Q5 (Figure 6c,d). Moreover, the EDS spectra analysis showed a high concentration of P, Ca, and Pb in the biomass of pure strain Q3 (Figure 8). Furthermore, under the same initial metal concentration, the presence of Pb(II) was higher in the biomass of strain Q3 than strain Q5. This result proved that the precipitation of Pb(II) as lead phosphate was induced by *Bacillus* sp. Q3. On the basis of EDS spectra analysis on experiments with 100mg L^−1^ of Pb(II) (Figure 8), Mg^2+^ was found in a higher amount in biomass of strain Q5 rather than strain Q3 and in precipitates of negative control tests, it is highly likely that the strain Q5 transferred Pb(II) to the intracellular region using Mg^2+^ transport system.

## 4. Discussion

### 4.1. Optimization of Pb(II) and Cd(II) Bioremediation Process

According to growth rate and metal bioremediation results, the new isolated bacterial strains Q3 and Q5 showed a different behavior with variations of initial pH: the growth rate is higher at pH 8, whereas Pb(II) and Cd (II) removal are better performed at a pH ranging between 5 and 6. The initial pH has an important role in the interactions between microbial cells and the chemical components of the environment [5]. In an alkaline environment, Pb(II) and Cd(II) are less soluble and therefore less harmful to the bacteria; as a result, the bacterial growth is not inhibited. According to the results of the growth rate, the optimal range of independent factors including initial pH, temperature, and initial metal concentration was found and defined to optimize the bioremediation process. In this study, the interaction between initial pH and temperature showed a significant effect on the bioremediation process performance for both Pb(II) and Cd(II). For instance, from the results of this study, it was evident that the Cd(II) removal efficiency increased when temperature increased and initial pH decreased. Several researchers reported that pH plays a critical role in the PTEs-remediation process [1,35,36]. Arivalagan et al. [37] found that the highest Cd(II) biosorption efficiency in *Bacillus cereus* strain KTSMBNL43 occurs at the following operating conditions: temperature of 35 °C, pH of 6.0, and initial Cd(II) concentration of 200 mg L^−1^, F, Choi et al. [38] found that the removal efficiency for Cd(II) in *Bacillus* sp. is higher at a pH above 3. Actually, Li et al. [1] studied the effect of pH and temperature on the Pb(II) remediation capability shown by *Rhodobacter sphaeroides*: they observed the highest remediation efficiency at a temperature ranging from 30 and 35 °C and pH 7. On the other hand, Hadiani et al. [35] reported that Pb(II) and Cd(II) were significantly removed by *Saccharomyces cerevisiae* at pH 6 and 5.5, respectively. The pH of the growth medium plays a key role in the availability of metal ions: at low pH (i.e., 5–6), the biosorption of metals on the microorganism cell wall could be higher because metals are more soluble [10]. Furthermore, temperature, as a regulator agent of bacteria metabolism, plays an essential role in controlling bacteria growth and, usually at a temperature between 30 to 40 °C, bacteria metabolism and enzyme activity achieve the highest rates. Hlihor et al. [39] found that the living biomass of *Arthrobacter viscosus* is capable of removing 96% of Pb(II) at a temperature of 28 ± 2 °C and pH of 6. Concerning the interaction observed between temperature and initial metal concentration, it can be reasonably asserted that Cd(II), at a concentration higher than 50 mg L^−1^, could strongly inhibit the growth of bacterial strains and consequently, their metal bioaccumulation potential. PTEs such as Cd(II) cause oxidative damage and reduce the bioremediation capacity of microorganisms [40]. Moreover, an increase in temperature induces an increase of the metals’ solubility, thus improving the accessibility of metals [41]. Concerning the results from mixed culture (i.e., Q3 + Q5) experiments and their higher efficiency in removing Cd(II) and Pb(II) rather than from pure cultures experiments, this aspect can be explained considering the synergic interactions between the two species that can generate strategies for enduring severe living conditions, and, furthermore, as other studies proved, mixed cultures have a higher probability to adapt to environmental changes than pure cultures [36,42].

Finally, according to optimal operating results, the highest Pb(II) and Cd(II) removal efficiency occurred at initial pH between 5 and 6, and temperature ranging from 35 and 38 °C. This result is in agreement with those reported in the manuscript of Roşca et al. [43], where the authors stated that *Bacillus megaterium* showed the highest performance in removing Cd(II) at acidic conditions and temperature of 35 °C. Moreover, results showed that the optimal initial pH varies accordingly to metal element types. Bacterial strains use different mechanisms to reduce the toxicity of PTEs in the environment, including bond formation between metal ions and the external part of bacteria cells [44,45]. Regarding this specific mechanism, pH is responsible for connecting the metals to the cell wall [2,5,46]. 

### 4.2. Mechanisms of Pb (II) and Cd (II) Bioremediation Used by Bacterial Strains Q3 and Q5

Microorganisms use different mechanisms to reduce the toxicity and mobility of PTEs [47]. Concerning the results obtained from FESEM and EDS analyses, it is reasonable to assume that the ions originally present on the surface of bacterial cells, such as Ca^2+^ and Mg^2+^, were involved in ion-exchanging process between Cd(II) and elements on the bacterial cells [28], and then Cd(II) penetrated the cell wall thanks to the Mg^2+^ transport system [48]. The presence of K^+^ and Mg^2+^ in the biomass of the bacterial strains, shown by EDS analysis, proved the occurrence of the ion-exchange mechanism and its involvement in the whole biosorption process between the bacterial cell and the investigated metals (i.e., Pb(II) and Cd(II)). Moreover, the compositions of the bacterial cell including carboxyl, hydroxyl, sulfate, and phosphate groups can affect the metals absorption process [2,49]. Indeed, Arivalagan et al. [37] found that the functional group C=O of the cell surface is mainly responsible for the Cd(II) biosorption process occurring in *Bacillus cereus*. Actually, in this study, *Bacillus* sp. Q5 bio-accumulated Cd(II) into the periplasmic space while the cell surface of *Bacillus* sp. Q3 was covered by Cd(II). Huang et al. (2018) [17] found that the *Bacillus cereus* RC-1 especially accumulates Cd(II) and Pb(II) inside the intracellular region. Furthermore, Cd(II) can precipitate on the surface of bacterial cells as insoluble crystals such as sulfate, phosphate, and carbonate, thus reducing the Cd(II) concentration in the environment and, consequently, its toxicity [50,51]. Besides, Kulkarn et al. [52] reported that adsorption, ion exchange, and ion complexation are the fundamental mechanisms associated with the Cd(II) biosorption process taking place in *Bacillus laterosporus*. In this study, the percentage of P and S increased with Cd(II) bioaccumulation in *Bacillus* sp. Q5, indicating that Cd(II) is precipitated as cadmium sulfate and cadmium phosphate.

Concerning the Pb(II) bioremediation process, lead resistant bacteria can utilize different mechanisms, including biosorption, efflux mechanism, induced precipitation, extracellular sequestration, and intracellular lead bioaccumulation [49]. In the presence of Pb(II), an influx transporter such as P-type ATPases can transfer and accumulate Pb(II) into the periplasmic space of the bacterial cell [27]. When Pb(II) concentration increased the resistance gene Pbr, as an efflux pump, transports and immobilizes Pb(II) on the cell surface as insoluble forms, thus reducing the toxicity induced by Pb(II) [49]. The uptake efficiency of PTEs such as Pb(II) as well as Cd(II) increased by inhibiting the ATPases activities [11]. During precipitation, microorganisms can convert the toxic metal ions into insoluble complexes such as phosphate, carbonate, and sulfate, thus reducing their concentration and consequently the toxicity of the contaminated sites [53]. According to results reported in this study, Naik et al. [53] demonstrated that the bio-precipitation of Pb(II) as lead phosphate occurred in *Providentia alcalifaciens* strain 2EA, whereas, De et al. [54] observed the precipitation of Pb(II) as insoluble lead sulfide on the cells of *B.iodinium* GP13 and *B. pumilus* S3.

## 5. Conclusions

In this study, two new strains of *Bacillus* sp. Q3 and Q5 were isolated from a sulfurous spring and their effectiveness for the bioremediation process was investigated using RSM with central composite design (CCD) statistical analysis by varying operating conditions in terms of initial pH, temperature, and initial Pb(II) and Cd(II) concentration. The results prove that the CCD design can be successfully used for optimizing the independent parameters in the metals bioremediation process. Moreover, pH was found to be an extremely important regulatory factor for the process as it strongly affects the mechanisms that concur to achieve the bioremediation process efficiency. The FESEM-EDS analysis showed that the bacterial strains Q3 and Q5 can bio-accumulate Cd (II) into the cell wall and induce the precipitation of Pb(II) as an insoluble crystal such as lead sulfate. The Mg^2+^ transport system is the most probable candidate mechanism that can be considered responsible for the bio-transfer of the metal ions. The results suggest that the bioremediation process conducted by *Bacillus* sp. strain Q3 and Q5 could be a valuable as well as an environmentally friendly and low-cost alternative to chemical and physical methods for reducing or even removing the toxicity of PTEs from contaminated sites.

## Figures and Tables

**Table 1:** Experimental independent variables.

Variables	Factor	Range and Level		
		−1	0	+1
Temperature (°C)	A	30	35	40
pH	B	5	6.5	8
Metal concentration (mg L^−1^)	C	50	125	200

**Table 2:** RSM experimental setup and results for lead (Pb) and cadmium (Cd) removal study.

Run no.	Variables			Removal Efficiency (%)											
	A	B	C	Q_3_				Q_5_				Q_3_ + Q5			
				Pb(II)		Cd(II)		Pb(II)		Cd(II)		Pb(II)		Cd(II)	
				Y _exp_	Y_pre_	Y _exp_	Y_pre_	Y _exp_	Y_pre_	Y_exp_	Y_pre_	Y_exp_	Y_pre_	Y _xp_	Y_pre_
1	40	6.5	125	92.07	93.29	35.58	32.60	66.29	66.68	48.41	46.82	73.63	71.75	37.13	37.62
2	35	6.5	125	87.53	89.51	32.02	32.00	75.37	75.45	48.64	48.40	86.08	85.74	43.08	42.42
3	40	8.0	200	19.78	20.60	12.97	10.97	24.92	24.04	20.70	20.17	31.13	34.19	29.90	28.52
4	30	6.5	125	71.90	71.10	17.86	18.72	69.85	70.51	30.12	31.85	70.18	70.34	24.52	25.82
5	30	8.0	50	31.32	30.74	8.95	6.67	25.27	22.64	16.66	15.46	26.32	28.51	16.08	14.59
6	40	5.0	50	56.00	55.21	57.73	58.21	35.38	35.38	77.54	77.51	33.69	35.82	68.11	67.23
7	35	6.5	125	90.17	89.51	30.82	32.00	76.58	75.45	47.54	48.40	87.08	85.74	43.62	42.42
8	30	8.0	200	10.05	10.73	5.81	5.85	24.07	23.99	16.04	16.39	44.53	42.82	27.51	27.94
9	40	8.0	50	36.55	34.82	19.67	21.35	16.29	14.60	32.07	33.06	25.36	23.85	28.50	29.22
10	35	6.5	50	69.48	73.51	40.52	38.09	55.93	59.83	57.60	57.35	65.31	65.07	46.66	47.37
11	35	6.5	125	89.51	89.51	31.93	32.00	73.26	75.45	48.82	48.40	85.23	85.74	42.24	42.42
12	40	5.0	200	65.34	65.82	17.61	20.42	37.10	39.46	24.75	25.91	41.78	40.02	29.16	30.20
13	30	5.0	50	21.61	20.69	33.04	35.57	42.29	42.90	50.87	51.36	26.90	24.32	43.28	44.21
14	35	6.5	125	89.98	89.51	32.93	32.00	75.56	75.45	48.96	48.40	83.64	85.74	44.50	42.42
15	30	5.0	200	23.87	25.50	8.50	7.34	37.65	39.08	14.60	13.58	30.61	32.54	22.39	21.23
16	35	6.5	200	72.40	68.80	18.47	18.78	65.49	62.65	31.63	32.02	75.80	74.33	34.45	35.53
17	35	5.0	125	67.87	67.48	44.97	40.30	64.43	60.21	55.46	54.86	63.63	63.90	52.33	52.39
18	35	6.5	125	91.22	89.51	30.49	32.00	75.90	75.45	48.53	48.40	85.10	85.74	42.01	42.42
19	35	6.5	125	89.52	89.51	29.58	32.00	78.14	75.45	48.17	48.40	83.86	85.74	42.66	42.42
20	35	8.0	125	49.07	49.89	18.57	21.12	37.10	42.37	33.30	34.04	65.04	63.06	35.00	36.73

**Table 3:** Analysis of variance (ANOVA) for the central composite design (CCD) model.

Metal Ions	Model Term	Tested Bacterial Strains								
		Q3			Q5			Q3 + Q5		
		DF	Mean Square	*p*-Value	DF	Mean Square	*p*-Value	DF	Mean Square	*p*-Value
Pb(II)	Model	9	1678.61	<0.001	9	966.04	<0.001	9	1192.88	<0.001
	A	1	1231.88	<0.001	1	36.67	0.086	1	4.97	0.374
	B	1	772.99	<0.001	1	795.66	<0.001	1	1.79	0.589
	C	1	55.32	0.007	1	19.80	0.193	1	214.09	<0.001
	AB	1	463.60	<0.001	1	0.056	0.942	1	130.57	<0.001
	AC	1	16.76	0.092	1	32.76	0.103	1	8.12	0.261
	BC	1	308.02	<0.001	1	13.39	0.278	1	18.54	0.102
	A^2^	1	147.10	<0.001	1	128.97	0.005	1	593.22	<0.001
	B^2^	1	2613.61	<0.001	1	1604.28	<0.001	1	1362.31	<0.001
	C^2^	1	926.86	<0.001	1	555.15	<0.001	1	707.28	<0.001
		R^2^ = 0.997			R^2^ = 0.988			R^2^ = 0.995		
Cd(II)	Model	9	363.48	<0.001	9	576.67	<0.001	9	290.89	<0.001
	A	1	481.52	<0.001	1	559.95	<0.001	1	348.30	<0.001
	B	1	919.22	<0.001	1	1083.13	<0.001	1	612.59	<0.001
	C	1	931.94	<0.001	1	1604.42	<0.001	1	350.66	<0.001
	AB	1	31.70	0.078	1	36.58	<0.001	1	35.21	0.002
	AC	1	45.73	0.040	1	95.51	<0.001	1	98.71	<0.001
	BC	1	375.63	<0.001	1	749.34	<0.001	1	660.17	<0.001
	A^2^	1	110.57	0.004	1	225.70	<0.001	1	314.77	<0.001
	B^2^	1	4.59	0.472	1	42.81	<0.001	1	12.60	0.037
	C^2^	1	35.01	0.066	1	37.88	<0.001	1	2.59	<0.001
		R^2^ = 0.975			R^2^ = 0.997			R^2^ = 0.992		

**Table 4:** Optimal operating conditions for the Pb(II) and Cd(II) bioremediation process conducted by bacterial strains Q3 and Q5 and their mixture (Q3 + Q5).

Metal Ions	Bacterial Strains	Temperature (°C)	Initial pH	Metal Concentration (mg L^−1^)	Removal Efficiency (%)
Pb(II)	Q3	38.8	5.8	115.4	93.8
	Q5	34.3	6.2	127.4	76.4
	Q3 + Q5	35.1	6.5	135.8	86.1
Cd(II)	Q3	38.6	5.0	50.6	58.0
	Q5	38.3	5.0	50.0	78.0
	Q3 + Q5	38.0	5.1	51.4	68.2
